# Developmental expression of BK channels in chick cochlear hair cells

**DOI:** 10.1186/1471-213X-9-67

**Published:** 2009-12-15

**Authors:** Yi Li, Graham M Atkin, Marti M Morales, Li Qian Liu, Mingjie Tong, R Keith Duncan

**Affiliations:** 1 School of Public Health, University of Illinois at Chicago, Chicago, Illinois, USA; 2 Neuroscience Program, The University of Michigan, Ann Arbor, Michigan, USA; 3 Kresge Hearing Research Institute, The University of Michigan, Ann Arbor, Michigan, USA

## Abstract

**Background:**

Cochlear hair cells are high-frequency sensory receptors. At the onset of hearing, hair cells acquire fast, calcium-activated potassium (BK) currents, turning immature spiking cells into functional receptors. In non-mammalian vertebrates, the number and kinetics of BK channels are varied systematically along the frequency-axis of the cochlea giving rise to an intrinsic electrical tuning mechanism. The processes that control the appearance and heterogeneity of hair cell BK currents remain unclear.

**Results:**

Quantitative PCR results showed a non-monotonic increase in BK α subunit expression throughout embryonic development of the chick auditory organ (i.e. basilar papilla). Expression peaked near embryonic day (E) 19 with six times the transcript level of E11 sensory epithelia. The steady increase in gene expression from E11 to E19 could not explain the sudden acquisition of currents at E18-19, implicating post-transcriptional mechanisms. Protein expression also preceded function but progressed in a sequence from diffuse cytoplasmic staining at early ages to punctate membrane-bound clusters at E18. Electrophysiology data confirmed a continued refinement of BK trafficking from E18 to E20, indicating a translocation of BK clusters from supranuclear to subnuclear domains over this critical developmental age.

**Conclusions:**

Gene products encoding BK α subunits are detected up to 8 days before the acquisition of anti-BK clusters and functional BK currents. Therefore, post-transcriptional mechanisms seem to play a key role in the delayed emergence of calcium-sensitive currents. We suggest that regulation of translation and trafficking of functional α subunits, near voltage-gated calcium channels, leads to functional BK currents at the onset of hearing.

## Background

A central feature in the maturation of hearing is a transition in the electrical signature of cochlear hair cells from spontaneous calcium spikes to graded receptor potentials [1,2]. The sequence of events associated with this transition are similar in mouse and chick [see for review, [3]], suggesting some commonalities between mammals and non-mammals as well as precocial and altricial animals. Immature hair cells, even shortly after terminal mitosis, exhibit several classes of essential ion channels, including mechanotransduction channels [4,5], voltage-gated calcium channels [6,7], and delayed rectifier potassium channels [2,8]. At this pre-hearing developmental stage, fast-activating, low-threshold calcium channels facilitate broad, slowly repetitive calcium action potentials. Spontaneous calcium spikes cease prior to the onset of hearing, due to the appearance of a slow voltage-gated potassium channel that drives down the hair cell's receptor potential and reduces the probability of calcium channel activity at rest [2]. Even so, calcium action potentials still can be evoked with depolarization. These calcium spikes are capable of promoting exocytosis [6,9] and post-synaptic activity [10], presumably for neurotrophic support and synaptic refinement.

Large-conductance BK-type potassium currents appear in hair cells at the transition from immature to mature excitability, coincident with the onset of hearing. Rapid activation kinetics and sensitivity to both depolarization and calcium enable BK channels to provide effective feedback on calcium influx upon hair cell stimulation. The presence of BK channels eliminates calcium spikes (spontaneous or evoked) associated with immature hair cells [2,11] and limits neurotransmission in mature hair cells [12,13]. At this point, the similarity in hair cell maturation in mammals and non-mammals diverges. In mammals, BK channels are primarily localized to the apicolateral membrane [14,15], distant from basolateral voltage-gated calcium channels present at synaptic active zones. The calcium source driving a negative voltage activation range in mammalian hair cell BK channels remains unclear, but activity is regulated by sources other than voltage-gated calcium influx [12,16,17]. In non-mammalian vertebrates, BK channels are colocalized with voltage-gated calcium channels and synaptic release sites [18,19]. The proximity of these channels contributes to an electrical resonance in the receptor potentials of hair cells from fish, frogs, alligators, and chicks [20]. Resonant frequency is systematically distributed along the auditory organ, corresponding to the sensitivity and selectivity of sound frequencies across the sensory epithelium and giving rise to an intrinsic electrical tuning mechanism [21,22]. Variations in BK channel kinetics contribute to the wide range of resonant frequencies necessary for encoding sound, leading to the surprising conclusion that the molecular structure of hair cell BK channels must also be systematically varied along the frequency axis of the cochlea [22-24]. Although the specific molecular underpinings of these functional effects remain unclear, alternative splicing of pore-forming α subunits and co-assembly with auxiliary β subunits have been proposed to play important roles [25-29].

The appearance of BK currents in several species, therefore, is tied to both hair cell maturation and frequency tuning, essential features of normal hearing. Even so, the mechanisms behind a late-stage appearance of BK currents at the onset of hearing are unknown. In the chick, these currents appear suddenly at E19 [11], when there is a substantial improvement in hearing thresholds [30]. In this paper, we describe the development of BK channel gene and protein expression in chick cochlea, in order to determine whether changes in gene transcription, protein translation, or subunit trafficking underlie the sudden acquisition of BK currents. Transcripts encoding the BK α subunit are present as early as E11, shortly after terminal hair cell differentiation [31,32]. Steady increases in transcript level up to E18 cannot explain the abrupt appearance of calcium-sensitive currents at that age. Transcriptional control of highly calcium-sensitive isoforms, including those incorporating the α subunit splice variant STREX and those co-assembled with β subunits, also cannot explain current acquisition at E18. In contrast, immunostaining reveals the formation of BK plaques beginning when currents are acquired. Thus, our results suggest that BK translation and trafficking are key regulatory events associated with the delayed acquisition of BK currents at the onset of hearing.

## Results

### BK gene expression in cochlear development

**Figure 1:**
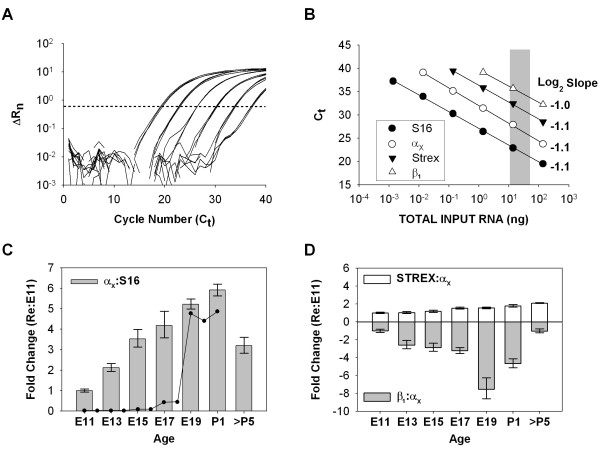
**The expression of BK transcripts during development of the chick auditory epithelium**. (A) Cycle-by-cycle amplification data are shown for S16 using serial dilutions of total RNA from chick basilar papilla. Threshold (dotted line) was manually drawn at a point during the log-linear phase of amplification. Cycle threshold was determined by identifying the threshold crossing-point for each amplification curve. (B) Total input RNA was serially diluted starting from a concentrated sample of chick basilar papilla. Diluted RNA was reverse transcribed and analyzed by qPCR. Efficiency data were fit by linear least-squares regression to C_t _= M*log_2_(dilution)+B. The slope (M) for each curve was between -1.0 and -1.1, indicating equal efficiency and 2-fold amplification per cycle. (C) The expression of BK transcripts, assessed by the non-variant-specific probe α_X_, gradually increased during late-stage maturation but decreased again in the first week after hatching. The time course for acquiring calcium-sensitive BK currents is illustrated (black dots) based on data from Fuchs and Sokolwski (1990). (D) The proportion of STREX splice variants among the total population of BK transcripts steadily increased during development, whereas the proportion of β_1 _decreased at the onset of hearing.

Real-time PCR was used to quantify the relative abundance of BK transcripts during late-stage cochlear development in the chick to test whether an upregulation of these transcripts near E19 could explain the acquisition of currents at this time point. TaqMan probes were developed for the pore-forming BK α subunit and the housekeeping gene S16 (Table 1). An example of the cycle-by-cycle QPCR fluorescence data is shown in Figure 1A for the S16 probe using serially diluted template. Reactions were run in triplicate and the data proved highly reproducible even for low-copy templates amplifying at later cycles. Efficiency curves were generated using serial dilutions of the total RNA samples (Figure 1B). This approach allowed us to test for amplification efficiency and for differential effects of reverse transcription depending on the amount of starting material. Amplification efficiency is determined by least-squares fits to the dilution curves using a log_2 _scale. A slope of -1.0 indicates an amplification efficiency of 100%. The slopes of the efficiency curves for α_X _and S16 were similar and near unity, validating the use of these probes for relative gene expression studies. QPCR products visualized on agarose gels were restricted to a single, specific amplicon of expected size (data not shown). Amplification curves for dissection saline and no-RT controls fell below threshold lines (undetected) or exhibited C_t _values far greater than the experimental samples (i.e. a difference of greater than 7 C_t_s or a reduction of more than 128-fold in starting material).

**Table 1:** Primer and probe combinations for real-time PCR.

Target	TaqMan^® ^Probe Sequence	Primers
**S16**	ACAGGTTCAAGCAGTTTGT	Forward: AATGATTGAGCCTAGGACTTTGCA Reverse: TCAACACCAGCAAATCTCTCCTT

α_X_	CATGAGCGCAACATAC	Forward: CAGCGTGCTGGACTCTCT Reverse: CAATGTCCGTATCAGTGTGAGGAT

**STREX**	TTTACGTGCCTTTGAAGATG	Forward: CTTCCCACTTTCTTCTGTCTCTGTT Reverse: GTGATAGTGTTGACGGCTGCT

**β_1_**	AAAGTGCTCGTATGTCCC	Forward: CACTGAAGATACCCTGGAAAGAAATCC Reverse: GTGATAGTGTTGACGGCTGCT

The expression of α_X _was normalized to the housekeeping gene S16 and reported relative to levels at E11 (Figure 1C). Positive values indicate fold-increases in transcript level. Gene expression steadily increased during these late embryonic ages, peaking in hatchlings at a level 6-fold greater than in E11. Expression levels decreased again as posthatch animals matured. The dependence on age was statistically reliable (one-way ANOVA, *p *< 0.01), but the stepwise changes in overall BK α expression is inconsistent with the sudden appearance of functional channels at the onset of hearing (black dots in Figure 1C). Another way to transcriptionally regulate functional currents is to specifically upregulate exceptionally calcium-sensitive isoforms at E18 - E19. Fourteen BK splice variants have been amplified or cloned from the chick cochlea [28,29] and additional variants have been identified in other species. Among these isoforms, inserts referred to as STREX-1 and STREX-2 have the greatest impact on apparent calcium affinity [27,33]. STREX-1 is a 58 amino acid insert in the C-terminus tail of the BK α subunit. STREX-2 is a concatenation of STREX-1 incorporating an additional upstream insert 3 amino acids in length. Both variants exhibit similar biophysical properties. A TaqMan probe was designed across the 3' splice junction between the 58 amino acid insert and the downstream constitutive exon, thereby targeting both STREX-1 and STREX-2 isoforms. The amplification efficiency for this probe was nearly identical to S16 and α_X _(Figure 1B, STREX). To determine whether there was an upregulation of STREX among the total population of BK α transcripts, the results were normalized to α_X _rather than the housekeeping gene S16. The representation of STREX among total BK transcripts steadily increased throughout development (Figure 1D), increasing by 2-fold in posthatch animals compared with E11 (one-way ANOVA, *p *< 0.01). Incorporation of the BK β_1 _subunit also increases BK calcium affinity [27,34], providing another mechanism for transcriptional control of hair cell BK currents. Although mRNA encoding β_1 _has been found in avian and mammalian cochleae [35,36], a role for this subunit in the physiology of mature hair cells remains unclear [37,38]. Nevertheless, it is possible that the developmental regulation of BK β_1 _could explain the sudden appearance of calcium-sensitive currents at E18-E19. Although the amplification efficiency for this probe was nearly 100%, the mean C_t_s typically fell between 35 and 38, suggesting that β_1 _transcripts were low-abundant. When normalized to α_X_, expression data showed a surprising decrease (up to 6-fold) in β_1 _coincident with the onset of hearing (one-way ANOVA, *p *< 0.01).

### BK protein expression in cochlear development

**Figure 2:**
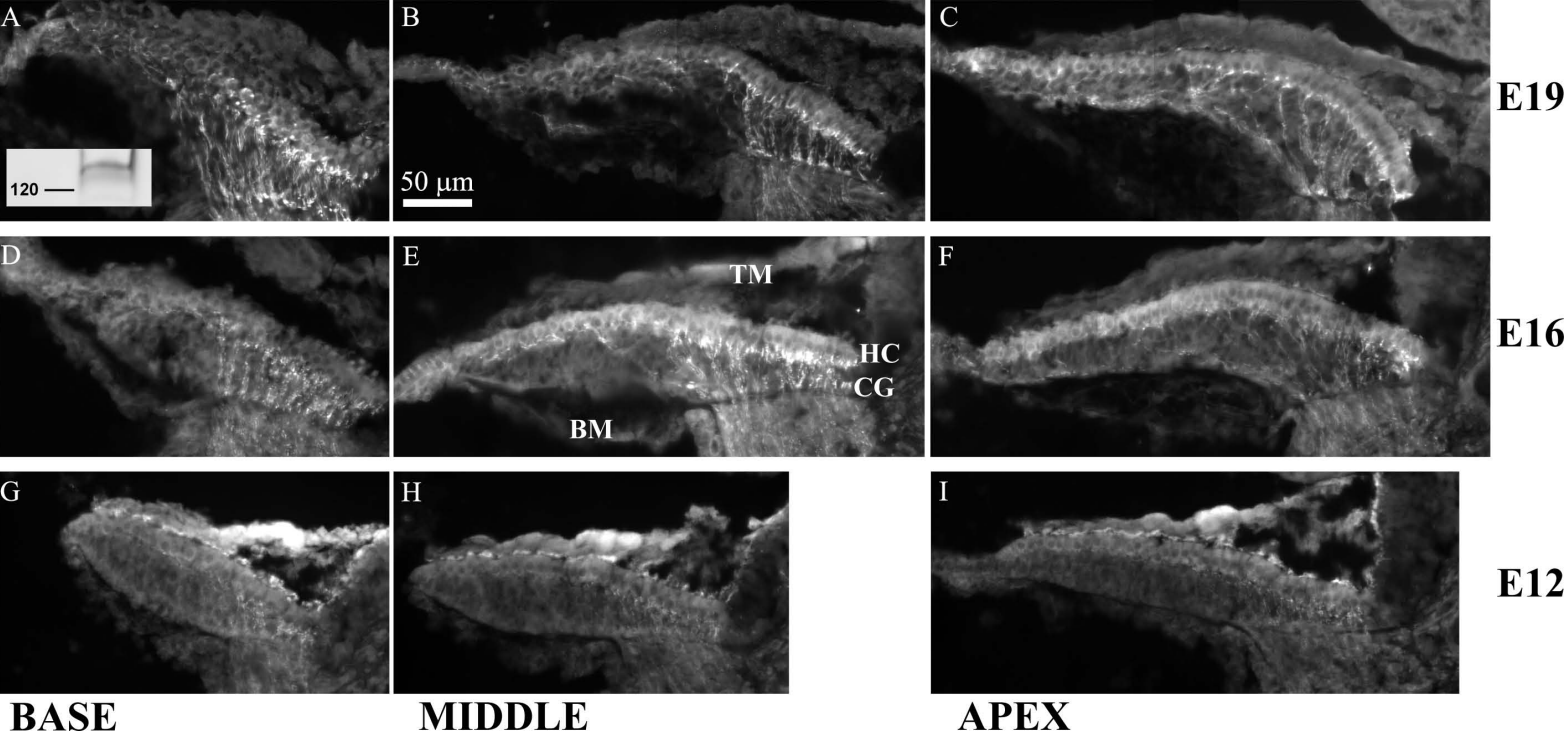
**The expression of BK subunits during development of the chick basilar papilla**. Cross-sections of the chick cochlear were stained with anti-BK (Chemicon) to qualitatively correlate protein levels with gene expression levels. Panels are organized by tonotopic position (base-to-apex, left-to-right) and age (E19-to-E12, top-to-bottom). Each cross-section is positioned so that the inferior to superior radial axis lies from left-to-right. Cochlear nerve projections were immunopositive as were the base of tall hair cells lying along the superior-most portions of most sections. Antibody specificity was confirmed using Western blot on membrane fractions from chick brain (inset in panel A). The dominant band appeared near 140 kDa, matching the predicted weight of the long C-terminus BK α variant. Additional bands near this molecular weight may be other splice forms or proteolytic by-products. No other spurious bands were observed in the blot. Scale bar in (B) is applicable to all panels. TM, tectorial membrane. HC, hair cell layer. CG, cochlear ganglion projections.

Developmental changes in α_X_, STREX, and β_1 _mRNA levels cannot account for the sudden changes in BK function. To determine whether a delay in protein translation could underlie the late appearance of currents, BK protein expression was analyzed immunohistochemically. Tall hair cells residing along the superior edge of the papilla are primarily innervated by afferent fibers and are the cells most associated with electrical tuning and BK function. Radial sections were cut through cryopreserved temporal bones and collected such that each slide had representative sections throughout the long, frequency axis of the cochlea. Sections were stained for anti-BK reactivity using a polyclonal antibody raised against a C-terminus fragment of the mouse α subunit (Chemicon anti-BK, see METHODS). Western blot analysis confirmed the specificity of this antibody to chick BK α subunits (Figure 2 inset). No-primary controls were immunonegative (data not shown). Exemplar images are shown in Figure 2 from basal, middle, and apical cross-sections of E12, E16, and E19 basilar papilla. Images were cropped to focus on the sensory epithelium, and each panel was arranged in a similar orientation for simplicity (i.e. inferior to superior, left to right). Immunopositive nerve fibers project from the cochlear ganglion (not shown) up to the base of the tall hair cells lining the superior-most portion of the hair cell layer. This staining was intense at E16 and E19 but faint at E12. A similar pattern was observed in several specimens. The strong anti-BK signal in cochlear projections at E16 coincides with late stages of synaptogenesis, extensive remodeling of peripheral synaptic endings [39], and maturation of ionic currents in the cochlear ganglion [40]. BK channels have been described in the mammalian auditory nerve [41], and there is evidence of voltage- and calcium-sensitive potassium currents in chick cochlear ganglion as well [40]. Antibody label was concentrated, often in a punctate pattern, at the base of the tallest hair cells, but it was difficult to distinguish between staining in the hair cell membrane and the nerve terminal.

**Figure 3:**
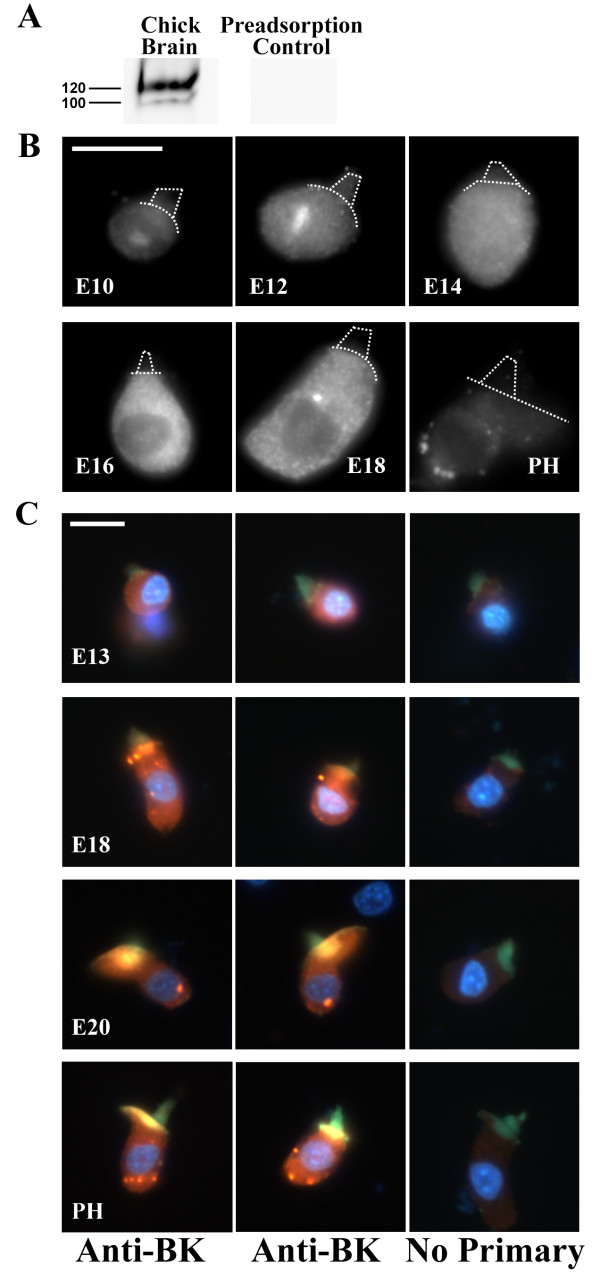
**BK channel clusters appear at E18 during hair cell maturation**. (A) Western blot analysis on membrane-bound protein from chick brain reveals a 120 kDa band, near the predicted weight of the minimal BK α variant (127 kDa; no inclusion of alternative exons). A fainter, lower-weight band is also apparent. No other spurious bands were observed in the blot. Both bands were absent when the primary antibody was preadsorbed with the immunogen. (B) Isolated hair cells from E10 to posthatch basilar papilla are shown labeled with antibodies to BK channels (Alomone). The apical surface and hair bundle are outlined to show orientation of the cell (dotted lines). Punctate clusters appear at E18. Bright label at E10 to E12 is inside the cell at the level of the nucleolus. (C) Several preparations were triple labeled for anti-BK (Alomone; red), hair bundles (phalloidin; green), and a nuclear stain (Hoechst; blue). Two examples for each age are shown (left two columns) along with no primary controls (far right column). Anti-BK clusters appear supranuclear at E18 and subnuclear from E20 onward. Scale bars = 10 μm.

To specifically investigate the emergence of anti-BK label in hair cells, immunocytochemical procedures were conducted on mechanically isolated hair cell preparations. Similar single-cell preparations have been used to identify the expression of BK channels in mammalian inner hair cells [14] and voltage-gated calcium channels in bullfrog saccular hair cells [42]. For these experiments, several polyclonal BK α antibodies were used with similar results (Chemicon and Alomone anti-BK, see METHODS). Data presented in the remainder of this report reflect staining from Alomone APC-107 anti-BK. The BK α subunit is highly alternatively spliced, and most antibodies--including the Chemicon antibody used in Figure 2--target epitopes that cross splice boundaries or include sequence from alternative exons. In contrast, the Alomone APC-107 anti-BK targets an epitope common to all splice forms. Specificity for the chick BK α homologue was confirmed using Western blot (Figure 3A). In chick cochlear hair cells isolated from E10 and E12 animals, the brightest anti-BK label was intracellular (Figure 3B). Confocal stacks clarified that this label was intranuclear. While surprising, this pattern is similar to that in chick ciliary ganglion at the same embryonic ages [43]. Diffuse cytoplasmic staining at E14 to E16 gave way to punctate staining from E18 onward. Punctate clusters in posthatch hair cells were located in subnuclear regions and appeared to be associated with the plasma membrane. Notably, the appearance of BK clusters coincided with the appearance of calcium-sensitive BK currents at E18-E19. These data strongly suggest that translation and trafficking of BK subunits into clustered domains, likely near voltage-gated calcium sources, underlie the delayed and sudden acquisition of BK currents during hair cell maturation.

These data also revealed an apparent translocation of BK puncta from supranuclear domains to subnuclear regions during development. To illustrate this in greater detail, additional isolated hair cell preparations underwent immunocytochemical analysis combining anti-BK immunofluorescence with markers for the actin-rich hair bundle and the nucleus, in order to clearly identify the position of BK clusters relative to these landmarks (Figure 3C). Again, no puncta were present at early embryonic ages (E13 shown, but similar results found for E10 through E16). Puncta appeared in supranuclear regions at E18. In contrast, BK puncta were concentrated in subnuclear regions from E20 onward. This pattern was replicated in several independent experiments and in numerous hair cells imaged at these ages. It is unclear whether the apparent movement of channels across the length of the cell reflects lateral diffusion of established plaques within the plasma membrane or *de novo *translation and trafficking of independent clusters.

The occurrence of BK-puncta was quantified across the developmental ages reported in Figure 3. In young embryos between E10 and E16, 0 of 47 cells (0%) exhibited anti-BK puncta. In late-stage embryos between E18 and E20, 16 of 27 cells (59%) exhibited one or more anti-BK puncta. In cells isolated from posthatch basilar papillae between P0 and P14, 45 of 53 cells (85%) showed punctate label. In 28 cells from P8 basilar papillae, the average number of puncta per cell was 2.2 ± 0.3, when counting only those spots twice the fluorescence intensity of background levels. The number of puncta per cell ranged from 0 to 6. Other spots differentially above background could be visually identified (0 to 4 per cell) but failed to meet the two-times-background criterion.

**Figure 4:**
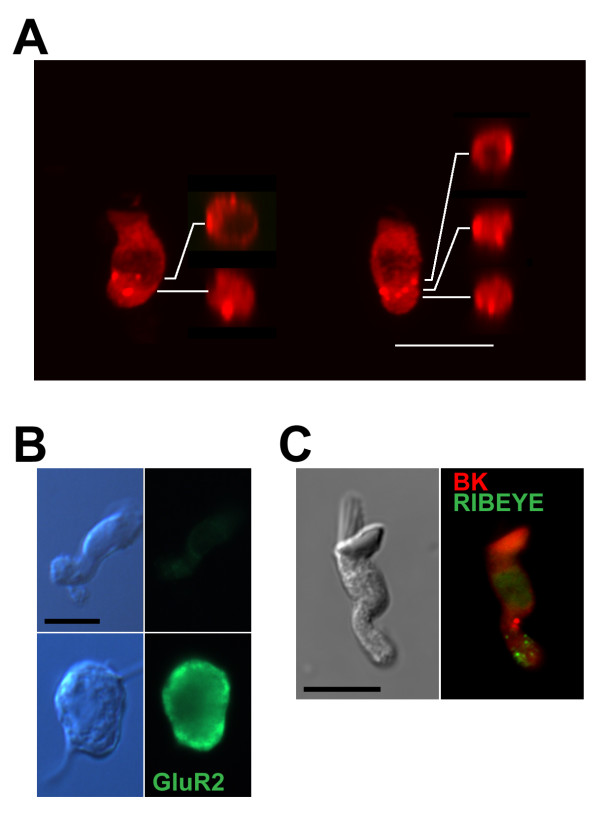
**BK channel clusters are associated with the hair cell membrane**. (A) Isolated hair cells were imaged using confocal microscopy to determine whether anti-BK clusters were associated with the membrane or found intracellularly. Two exemplar hair cells are shown, both oriented with hair bundles toward the top of the image. Between 13 and 17 optical sections were taken at increments of 0.5 μm. Projections of full Z-stack are shown alongside orthogonal cuts through the reconstructed stack. Cuts were made at the level of each anti-BK puncta and positioned to the right of each hair cell example. In all cases, puncta were present along the outer edge of the cut-view, indicating overlap with the hair cell plasma membrane. (B) Hair cells and auditory neurons were acutely isolated onto the same slide in some preparations and stained for glutamate receptor 2 (GluR2) in order to detect residual afferent terminals on the dissociated hair cells. Afferent auditory neurons were identified according to cell size and the presence of bipolar axonal projections. Under epifluorescence and using constant exposure conditions, anti-GluR2 label was found on the cell soma of auditory neurons but was absent from hair cells. (C) Anti-BK puncta were distant from synaptic ribbons in isolated hair cells. Isolated hair cells were co-labeled with anti-BK (red) and anti-ctbp2/RIBEYE (green), a marker for presynaptic ribbons. An exemplar tall hair cell is shown using epifluorescence imaging. Debris that might be associated with afferent terminals is absent from the matching differential interference contrast image (left). The image shown is representative of over 5 tall hair cells imaged in this co-labeling experiment. Scale bars in all panels = 10 μm.

Localization of anti-BK puncta to the surface of the hair cell membrane was supported by confocal microscopy (Figure 4A). Cuts through reconstructed Z-stacks showed that puncta were always positioned along the membrane border rather than internally (see also confocal movies in Additional files 1 and 2). Based on the neuronal labeling in Figure 2, it was possible that BK puncta reflected residual post-synaptic terminals rather than pre-synaptic clusters in the hair cell membrane. To address this possibility, we labeled isolated hair cells with pre- and post-synaptic markers to identify prevalence of post-synaptic terminals in our preparations and to determine the relationship between BK puncta and the synaptic active zone. Anti-GluR2 staining was negative on dissociated cells (Figure 4B), suggesting that terminals were adequately removed during enzymatic treatment and mechanical dissociation. Co-label experiments with antibodies to BK channels and the synaptic ribbon protein RIBEYE (anti-ctbp2) revealed a dislocalization of BK clusters and active zones (Figure 4C). Staining for the ribbon synapse can be considered a proxy for the location of synaptic active zones, even though the postsynaptic membrane covers a larger surface area than that marked by the ribbon itself [44,45]. For BK plaques to be associated with terminals, the postsynaptic footprint would have to be large enough to encapsulate both BK and ribbon puncta. Such a large amount of extraneous membrane would be visible under DIC optics. However, images in Figures 3 and 4 were taken from hair cells exhibiting a smooth surface. These data support an association between BK plaques and the hair cell membrane, and they challenge the widely held view that BK channels and voltage-gated calcium channels are clustered at presynaptic active zones in non-mammalian hair cells [18,19]. This observation requires further study, but it is possible that channel density at active zones is below our detection limits.

### Functional correlate of a delay in BK surface expression

**Figure 5:**
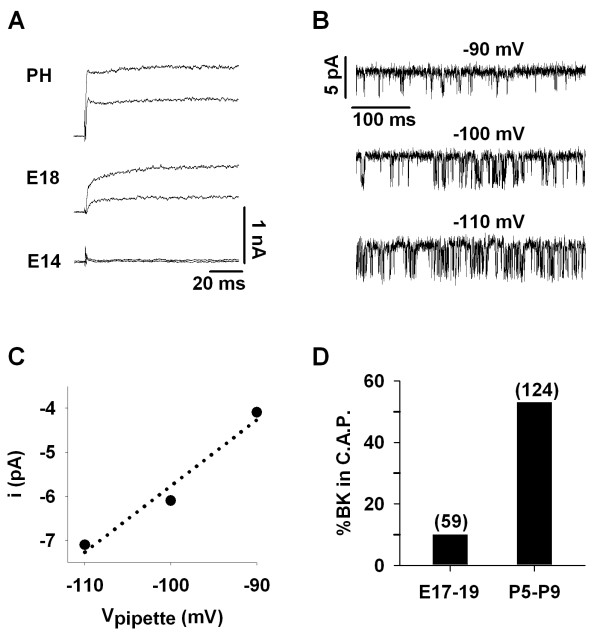
**The density of functional BK channels at the basolateral pole increases with age**. (A) Whole-cell recordings were made from embryonic and posthatch hair cells to confirm the onset of BK currents around E18/19. Example traces from three ages are shown. In these examples, currents were elicited by voltage steps from -80 mV to -20 or 0 mV. A fast-activating outward current was present at E18 under whole-cell recording conditions, but no such current could be recorded from earlier ages. (B) Single-channel current traces are shown for a cell-attached patch from a posthatch hair cell held at three different pipette potentials. Bath and pipette salines consisted of standard ECF and high potassium electrode solutions, respectively. Channel openings are downward. In this configuration, more negative pipette voltages correspond to increasing depolarization. The channel was voltage-sensitive, as evidenced by an increased open probability for more depolarized voltages. (C) Single-channel current amplitudes were estimated from all-points histograms using traces in (B). Linear regression to these data (dotted line) indicates a unitary conductance of 150 pS. Reversal was estimated to be about -60 mV, near the presumed resting potential of the cell. Therefore, all inclusion criteria were satisfied, establishing the identity of this channel as BK. (D) The percent of cell-attached patches (C.A.P.) exhibiting channel fluctuations attributed to BK are plotted for cells from embryonic and posthatch basilar papilla. The total number of attempts is shown in parentheses. The breakdown for the E17-E19 data set was: E17 (6%), E18 (7%), E19 (17%).

The magnitude of calcium-sensitive BK currents in the development of the chick basilar papilla has been quantified previously [11]. In that study, BK current substantially increased between E18 and E19, from little measurable calcium-sensitive current at the younger age to maximum levels only one day later. Since embryogenesis is highly dependent on flock age and health, incubation temperature, air flow around the egg, and humidity levels [46], we confirmed the time course of BK functional development using whole-cell voltage-clamp recordings. To isolate these currents, 15 mM 4-AP was included in the intracellular (electrode) solution to block slowly-activating delayed rectifier potassium channels [16]. The remaining fast-activating current was attributed to BK, and this was subsequently verified by removing extracellular calcium, leading to the elimination of the fast current (data not shown). Cells from animals at E18 and older exhibited this fast-activating, calcium-sensitive current, but cells from earlier ages showed no BK current, as reported previously (Figure 5A). The average steady-state currents measured during voltage steps to 0 mV were 652.1 ± 77.4 pA for posthatch (N = 5), 342.5 ± 69.2 pA for E18 (N = 4), and 27.7 ± 14.7 pA for E14 (N = 5) (mean ± SEM). All recordings were obtained from tall hair cells located 20% to 50% from the apical end of the papilla. These currents may be contaminated by small amounts of residual delayed rectifier channels, but the presence of a fast-activating current suggests that clustered channels at E18 are functional and sensitive to extracellular calcium.

The developmental time course for this current not only reflects changes in BK channel surface expression but also any change in the density of plasmalemmal calcium channels, the colocalization of BK and calcium channels, and intracellular calcium buffering. To assess changes in BK channel density specifically, the basolateral surface of tall hair cells was scanned for BK fluctuations using cell-attached patch recordings. In this way, large depolarizations could be used to detect the presence of BK channels in the membrane, regardless of the local intracellular calcium concentration or the apparent calcium affinity of the channel itself. An example of cell-attached patch records is shown in Figure 5B. In this patch, currents flickered between a closed current baseline and a single open-channel current level, indicating the presence of one channel in the patch. All-points histograms were generated to estimate changes in open probability and single-channel current amplitude. For the example in Figure 5B, open probability increased with more negative pipette potentials (i.e. increasing depolarization in this recording configuration). Single-channel conductance was estimated to be about 150 pS (Figure 5C), consistent with large-conductance BK channels under these recording conditions. Reversal occurred at approximately -60 mV (pipette command), indicating that the channel was potassium selective. Repeated attempts at cell-attached patch recordings in embryonic cochleae resulted in sporadic observations of BK-mediated current fluctuations. To systematically investigate this observation, we recorded the proportion of patches containing BK channels in hair cells from E17-E19 embryos and P2-P9 posthatch animals. The presence of BK currents in cell-attached patches was noted if single-channel fluctuations were (1) voltage dependent, (2) K^+ ^selective, and (3) large conductance (see METHODS). Using these criteria, only 10% of patches from E17-E19 hair cells included BK channels whereas over 50% of patches from posthatch animals exhibited BK-related fluctuations (Figure 5D). Since the patches were obtained from the basal pole of hair cells, these results support the conclusions drawn from the immunocytochemistry data in Figure 3, showing an apparent translocation of BK channels from supranuclear to subnuclear regions.

## Discussion

Calcium-dependent BK current appears at the onset of hearing in hair cells of both mammalian and non-mammalian species, but the mechanisms underlying acquisition of this important current remain unknown. Transcriptional upregulation of BK-related mRNA would be the simplest explanation for sudden changes in function. However, our results demonstrated a steady increase in BK mRNA during maturation of the chick cochlea, increasing only 6-fold over the week prior to hatching. Moreover, developmental changes in the mRNA levels of highly calcium-sensitive subunits were unable to explain functional changes. Considerable effort has been expended in the past decade to correlate mRNA levels with protein expression in a variety of systems [see for example, [47-49]]. The results clearly illustrate a nonlinear and dynamic progression from gene expression to protein function, indicating that mRNA analysis is an insufficient predictor of protein expression levels and functional effects [50]. These studies argue for a combined approach, focusing on both gene and protein expression. While it remains possible that upregulation of other splice variants contributes to a rapid functional time course, our results show a close correspondence between the acquisition of BK currents at E18-E19 and the appearance of anti-BK clusters in the basolateral membrane, implicating translation, trafficking, and scaffolding processes as rate-limiting steps in BK function.

Although BK channels are eventually clustered in different regions of the plasma membrane in mammals and non-mammals, the developmental sequence of anti-BK reactivity is strikingly similar across species beginning with diffuse cytoplasmic staining at early ages and ending with plaque formation at the onset of hearing. In mouse, cytoplasmic label was found as early as E18 [14], shortly after terminal differentiation of the hair cell in this species [51]. Similarly, we found immunopositive reactions in isolated chick hair cells as early as E11 in chick, only several days after terminal mitosis [31]. Diffuse cytoplasmic staining was accompanied by more intense label within the nucleus. Although it is tempting to dismiss this label as nonspecific, the pattern mirrors that described in ciliary ganglion [43] so the presence of full-length subunits or truncated peptides may represent an unknown dual-use of *KCNMA1*. There is mounting evidence for such multifunctional genes [52]. For example, the synaptic ribbon protein RIBEYE and the transcriptional repressor CTBP2 are encoded by alternative splicing in the same gene [53], leading to both nuclear and active zone immunolabel when using antibodies directed against the common C-terminal domain of RIBEYE/CTBP2 [54]. Dual uses of ion channel gene products also have been reported. Posttranslational cleavage of the L-type voltage-gated calcium channel CaV1.2 results in a C-terminal fragment that is capable of nuclear translocation and regulation of gene transcription [55].

Two distinct BK currents have been recorded in mammalian inner hair cells [16], including major and minor components that are, respectively, insensitive and sensitive to calcium influx through voltage-gated calcium channels. Immunolocalization of BK channels in these hair cells has revealed significant extrasynaptic labeling at apicolateral domains [15,56]. However, there is evidence for dot-like basolateral expression using both antibody staining and fluorescently tagged BK toxins [14]. Although direct evidence is lacking, the basolateral and apicolateral expression of BK may correlate with calcium sensitive and insensitive current components at synaptic and extrasynaptic locations, respectively. In the current study, colabeling experiments combining anti-BK with a marker for synaptic active zones (RIBEYE/CTBP2) revealed extrasynaptic expression in chick as well. This result was surprising based on indirect empirical evidence and theoretical models of electrical tuning that all support the colocalization of voltage-gated calcium channels and BK channels at synaptic active zones in lower vertebrate hair cells [18,19,57,58]. Our results could arise from lateral diffusion of BK plaques within the plasma membrane after hair cell isolation, but such diffusion is unlikely since the cells are fixed with cross-linking aldehydes within minutes after isolation. Alternatively, presynaptic BK channels may occur at a low density below our detection limits. These issues must be resolved to fully understand calcium signaling at the hair cell synapse and the relationship between this signaling and electrical tuning in non-mammalian vertebrates.

BK immunoreactivity was detected as early as E10 in the soma of chick auditory neurons (data not shown) and in afferent processes as early as E16, indicating that remodeling and trafficking of BK subunits occurs in auditory nerve, as well as sensory hair cells, during late stages of functional maturation. This is the first description of BK channels in axonal projections of auditory neurons, and these data suggest an influence on postsynaptic signaling. Nevertheless, the presence of BK channels in both hair cells and afferent projections raises the possibility that BK puncta on isolated hair cells arise from postsynaptic membrane fragments. However, several pieces of evidence support an association of these puncta with the hair cell membrane: (1) Images were selectively taken from cells exhibiting a smooth, birefringent membrane devoid of obvious postsynaptic footprints, (2) BK puncta first appear in isolated cells at E18, several days after label is seen in dendritic projections, (3) GluR2 labeling was negative on hair cells, (4) anti-BK puncta were distant from synaptic active zones where postsynaptic membrane fragments are most likely found, and (5) the translocation of BK clusters to the base of the cell between E18 and PH mirrors a change in the prevalence of BK in cell-attached patches.

Surface expression of clustered, functional BK channels is an essential feature in hair cell maturation, but the mechanisms involved in these processes remain unknown. Further, it is unclear whether surface expression, scaffolding into clusters, and trafficking to distinct microdomains is a sequential process or a simultaneous, coordinated event. For example, diffuse antibody label at early embryonic ages in chick (E10-E16) appeared cytoplasmic, but it is also possible that a portion of this immunoreactivity included membrane bound protein. Previous studies have identified several mechanisms for adjusting the density of these channels in the plasma membrane, including the expression of dominant negative, cytoplasmically retentive isoforms and co-expression with regulatory chaperones. Retentive BK α isoforms include the alternatively spliced C-terminus variant ending in VEDEC [59,60] and a novel variant (SV1) containing the retention signal CVLF [61,62]. Variants containing VEDEC have been described in rat and chick cochlea [29,35], but variants incorporating the CVLF motif have yet to be described in the auditory periphery of either species. In addition, a 4 amino-acid variant in the cytoplasmic tail of the BK α subunit contains a canonical ER retention signal (RKR). While this variant alters the biophysics of the channel, there is currently no evidence that this isoform alters surface expression in native tissue or heterologous expression systems [27,29,63]. Interestingly, intracellular retention of VEDEC can be rescued by a variety of chaperones [59,64,65]. Therefore, surface expression may be tied to transcript levels of specific isoforms as well as co-assembly with interacting proteins.

A compendium of BK channel interaction partners is emerging from yeast-two-hybrid screens, reciprocal co-immunoprecipitation studies, and mass spectrometry approaches [65-70]. The most complete study of the BK interactome identified partners classically associated with an array of cellular processes, including metabolism, trafficking/scaffolding, differentiation, signaling, transport, and transcription/translation [67]. In that study, more than 150 potential interactors were identified with a co-immunoprecipitation strategy. The BK α antibody used to pull-down complexed subunits was a commercially available polyclonal antibody raised against the C-terminus splice form ending in VEDEC. As a result, the study may have identified only a subset of candidate molecules. It remains to be seen whether additional interactors might be found using antibodies specific to other mutually exclusive C-terminus splice variants. Even so, over 20% of the candidate partners identified by Kathiresan et al. (2009) were involved in trafficking and scaffolding. Molecules involved in scaffolding proteins in macromolecular complexes include β-catenin [71], RACK1 [66], MAGI1 [69], caveolin [72], Lin7c [67], and the L-type voltage-gated Ca^2+ ^channel β subunit [73]. Several of these interactors are ideal candidates for colocalizing BK channels and voltage-gated calcium channels at either extrasynaptic or synaptic domains.

## Conclusions

In this study, we sought to combine mRNA expression data and protein expression studies to identify the key events underlying a late acquisition of BK channel currents in hair cell development. The results implicate regulation of BK trafficking in this process, surprisingly to extrasynaptic domains. However, it is unclear whether this process is a purely post-transcriptional event. Transcriptional control of scaffolding or chaperone elements could still underlie the sudden appearance of BK current at the onset of hearing, possibly tied to the cessation of spontaneous calcium spikes prior to the onset of hearing. In future experiments, it will be important to interfere with transcription, translation, and BK interactors in order to identify the relevant mechanisms.

## Methods

### Tissue preparation

All animal procedures were approved by the University of Michigan Committee on Use and Care of Animals. Cochlear preparations were obtained from embryonic and posthatch White Leghorn chickens (*Gallus gallus*). Posthatch chicks, from 1 to 14 days old, were anesthetized by intramuscular injection with approximately 40 mg/kg ketamine and 10 mg/kg xylazine and euthanized by rapid decapitation. The middle-ear cavity was exposed by removing extraneous soft tissues and opening the external auditory canal. In this manner, the base of the bony auditory duct was revealed, and the cochlea was easily extracted through the oval window. Embryos were staged according to Hamburger and Hamilton (1951), using the length of the 3^rd ^toe as an indicator of true developmental age. Fertilized eggs were obtained from Bilbie Aviaries (Ann Arbor, MI) or the Michigan State University Poultry Teaching and Research Center (East Lansing, MI) and maintained in a temperature and humidity controlled incubator. Embryos were euthanized by rapid decapitation. The cochleae of late stage embryos (E17 to E20) were harvested in the same manner as posthatch animals. The cartilaginous temporal bones of animals younger than E17 were placed directly into extracellular fluid (ECF) (in mM: 154 NaCl, 6 KCl, 5 CaCl_2_, 2 MgCl_2_, 5 HEPES, buffered to pH 7.4 with NaOH), and the cochlea was gently dissected from the auditory duct.

Extracted cochleae were immediately placed in ECF supplemented with protease (Type XXIV, Sigma) to facilitate further microdissection. Enzyme concentration (0.01% to 0.1%) and digestion time (1 to 10 minutes) were adjusted according to age, with the harshest conditions reserved for the earliest ages when cell adhesion appeared to be strongest. Following protease treatment, the cochlea was pinned in a dish coated with Sylgard (Dow Corning, Corp). The tegmentum vasculosum (analogous to the mammalian stria vascularis) and tectorial membrane were removed with forceps, and the sensory epithelia (i.e. basilar papilla, consisting of hair cells and interdigitating supporting cells) was gently teased from the underlying basilar membrane.

### Real-time PCR

Total RNA was extracted from the sensory epithelia of 6 to 12 cochlea using an RNeasy Micro Kit (Qiagen) or PicoPure RNA Isolation Kit (Molecular Devices) under RNase free conditions. Care was taken to obtain the entire length of the papilla to avoid biasing the gene expression data if regional variations in transcript levels were present. Following the collection of each pooled sample, an equivalent amount of dissection saline was reserved to test for possible contamination by RNA from disrupted accessory tissues. Pooled tissue samples and reserved saline were kept at -80°C for no more than one week before RNA extraction. The quantity and quality of the RNA from tissue samples were evaluated using an Agilent 2100 Bioanalyzer (Agilent Technologies). Samples were included in the study if the ratio of 28S:18S rRNA was >1.0. Reverse-transcription was accomplished using oligo(dT) primers and Superscript III (Invitrogen) according to manufacturer's instructions. Controls included samples for which the reverse-transcription enzyme was omitted from the reaction (no-RT controls).

Four to eight biological repeats were subjected to quantitative real-time polymerase chain reaction (QPCR) using TaqMan fluorogenic probes and commercially available PCR master mix (Applied Biosystems, Inc.). Primer and probe sequences for the custom TaqMan assays were developed in conjunction with Applied Biosystems, Inc. Probes were designed to cross splice junctions to ensure specific hybridization to reverse-transcribed cDNA rather than genomic DNA. Custom assays were developed according to published sequences or those derived from analysis of the chick genome. Sequences are shown in Table 1 for the reference housekeeping gene *Rps16 *(S16, encoding a component of the small 40S ribosomal subunit) [accession GenBank:XM_416113.2], *Cslo1 *(α_X_, encoding the pore-forming α subunit of the chick BK channel) [GenBank:NM_204224.1], the STREX splice variant of *Cslo1 *[GenBank:AF076268.1], and the chick BK β_1 _gene [GenBank:NM_204602.1]. The primer/probe combination targeting α_X _was specifically designed to cross a constitutive exon-exon boundary for which there is no known splice variant in order to capture all possible BK α transcripts. Therefore, this probe is considered a pan-BK α probe, capable of detecting all BK α mRNAs regardless of splice variation. Relative gene expression was determined using the ΔΔ*C*_*t *_method [74]. In this method, the accumulation of PCR products is monitored in real-time, allowing the placement of a threshold line in the log-linear phase of amplification. The PCR cycle at which the fluorescence reported by TaqMan chemistry crosses this threshold is referred to as the cycle threshold (*C*_*t*_). All reactions were run in triplicate and the mean *C*_*t *_for each triplicate set was recorded. Data were excluded if the standard deviation of triplicate *C*_*t*_s exceeded 0.3. Two normalizations are required to get the ΔΔ*C*_*t *_value used for relative expression comparisons. First, the gene of interest is related to a reference gene to obtain ). Next, the Δ*C*_*t *_of one experimental group is normalized to a reference group to obtain ). For example, to determine the change in α_X _in posthatch animals (PH) compared with E11 animals, the gene of interest (α_X_) is first normalized to the reference housekeeping gene S16 and then compared between the two age groups. The calculation would be as follows:. The relative fold change in gene expression is then calculated as . Reactions were run on an ABI Prism 7000 or 7900HT Sequence Detection System (Applied Biosystems, Inc.). A one-way analysis of variance (ANOVA) was used to determine the statistical reliability of gene expression changes during cochlear development (*p *< 0.05).

### Western blot on membrane fractions

Membrane fractions were obtained from posthatch chick brain (P14-P21) to validate BK antibodies. Approximately 30 mg of adult chick brain was collected and homogenized with a tissue tearor in 100 μl MES buffered saline (MBS) containing 1% Triton X-100 and 0.1% ABS-14. The homogenate was separated on discontinuous sucrose gradient by ultracentrifugation (200,000 × g in an Optima Max-E Ultracentrifuge (Beckman-Coulter) using a swinging bucket rotor (TLS-55) for 16-24 h at 4°C). Equal volume fractions were collected, mixed with Laemmli sample buffer containing 5% 2-mercaptoethanol, separated by SDS-PAGE, and transferred to nitrocellulose membranes. Antibodies used for Western blot analysis included purified polyclonal BK antibodies (1:500 APC-107, Alomone Labs, and 1:200 AB5228, Chemicon) and polyclonal anti-caveolin (1:2000 610060, BD Transduction Laboratories). Membrane fractions were first identified by immunoreactivity to anti-caveolin. These fractions were used to confirm BK antibody specificity. To conduct Western blot reactions, transferred proteins were blocked with 5% non-fat milk in TTBS (0.1% Tween 20 in 1× TBS) for one hour before applying primary antibodies overnight. After washing in TTBS, blots were incubated with secondary antibodies (1:5000 HRP-conjugated goat anti-rabbit) diluted in TTBS containing 5% non-fat milk and 0.1% BSA for one hour. Reactions were visualized with ChemiGlow West ECL reagent (Alpha Innotech) and detected using a FluorChem SP chemiluminescent imaging system (Alpha Innotech).

### Immunohistochemistry

Cryosections were obtained from embryonic and posthatch temporal bones. Freshly dissected temporal bones were submerged for 3 hours in room temperature paraformaldehyde (2%, diluted in 0.12 M phosphate buffer (PB)). Preparations from posthatch and late stage embryos were decalcified for 1 to 3 days with 5% EDTA in 0.12 M PB. Samples were infiltrated with 30% sucrose overnight, placed in an embedding mold filled with Tissue-Tek O.C.T. freezing medium (Sakura), and frozen in liquid nitrogen cooled isopentane. Serial sections 10 to 12 μm in thickness were alternately collected on 20 Superfrost Plus slides such that a single slide contained sections throughout the entire basilar papilla. Slides were stored at -80°C until required. Prior to immunofluorescence reactions, frozen slides were rehydrated with phosphate buffered saline (PBS) and blocked for 1 hour in 0.2% Triton X-100 (Calbiochem) with 5% normal goat or donkey serum. Sections were incubated with primary antibodies (Chemicon rabbit polyclonal anti-BK, AB5228) at 1:200 overnight at 4°C. The Chemicon anti-BK was derived using a GST-fusion protein consisting of the C-terminus of the mouse Slo1 homologue (Accession A48206, amino acid residues 1098-1196). The immunogen crosses a commonly reported C-terminus splice site, such that approximately two-thirds of the immunizing peptide is splice variant specific. Cross-reactivity to various BK isoforms remains to be verified. Immunoreactions were visualized with fluorescently conjugated secondary antibody (highly cross-absorbed AlexaFluor 594 goat or donkey anti-rabbit, Invitrogen) applied at 1:500 for 2 hours at room temperature. Tissues were prepared and imaged in parallel under the same staining and exposure conditions.

Isolated hair cells were obtained from embryonic and posthatch basilar papilla by mechanical trituration through a glass capillary pulled to approximately 100 to 300 μm in diameter. Cells were initially dispersed into ECF droplets contained within rubber-cement boundaries on ethanol washed Superfrost Plus microscope slides (Fisher Scientific). In some cases, slides were pre-coated with poly-l-lysine to improve cell adherence. Tens to hundreds of isolated cells could be obtained from a single basilar papilla. Cells were prepared for immunofluorescence by fixation in 4% paraformaldehyde for 15 minutes at room temperature, followed by permeablization in 0.1% Triton X-100 and blocking in 5% normal goat or donkey serum supplemented with 1% BSA diluted in PBS. Primary antibody (Alomone rabbit polyclonal anti-BK, APC-107) was applied at 1:400 for 3 hours at room temperature or 1:1200 overnight at 4°C. The Alomone antibody was raised against a synthetic peptide corresponding to a sequence within the cytoplasmic tail of the mouse Slo1 homologue (Accession Q08460, amino acid residues 1184-1200). This sequence is adjacent to, but does not cross, an exon-exon boundary. Therefore, the antibody is expected to cross-react with all BK α splice-forms. Positive label was detected with fluorescently labeled secondary as above. Isolated cells were counterstained with 488 AlexaFlour conjugated phalloidin (Invitrogen) and Hoechst 33242 (Invitrogen) to visualize hair cell hair bundles and nuclei, respectively. In both cryosections and isolated cell preparations, nonspecific secondary staining was evaluated in experiments omitting the primary antibody (no-primary controls).

Histological preparations were observed using a Leica microscope (DM LB) outfitted for differential interference contrast and epifluorescence microscopy. Images were digitized using an attached CCD camera (Micropublisher, QImaging) under the control of QCapture software (QImaging). Confocal images were obtained using a Zeiss LSM 510-META laser scanning confocal microscope. Projections and cuts through reconstructed Z-stacks were generated using Zeiss LSM Image Browser Software. Final images were prepared in Photoshop CS2 without manipulation beyond cropping and rotating.

The number of anti-BK plaques per cell was quantified using Metamorph (Molecular Devices). Images were obtained under constant exposure conditions. Puncta were counted if the fluorescence intensity of the spot was twice that of the average diffuse label within the cell. To be counted as puncta, these bright spots also had to appear associated with the plasma membrane and span1 μm or more in diameter. Cells were included if the long axis of the cell was greater than the cell width (i.e. a tall hair cell), the membrane exhibited a smooth appearance devoid of debris, and the cell was sufficiently isolated from supporting cells and other hair cells.

### Electrophysiology

Using fine minutien pins, serial segments of the sensory epithelium were isolated, transferred to recording chambers filled with ECF, and mounted under pins with hair bundles directed downward, as described previously [24,38]. In this configuration, the basolateral pole of the hair cell was made visible by suction of supporting cells, enabling electrode access to subnuclear portions of the hair cell membrane. Voltage-clamp recordings were made using an Axopatch 200B amplifier with a Digidata 1200 or 1322A digitizer (Molecular Devices, Sunnyvale, CA). Data were acquired using pCLAMP 9 software (Molecular Devices). The external solution consisted of ECF with 8 mM glucose. Glass electrodes were pulled from borosilicate capillaries (1B100F-4, World Precision Instruments, Sarasota, FL) to achieve an electrical resistance of 3-7 MΩ in the whole-cell recordings and 5-9 MΩ in patch recordings. Electrodes were coated with Sylgard or R6101 (Dow Corning) to reduce stray capacitance. BK channel fluctuations were observed in cell-attached and inside-out recording configurations [75]. In both cases, electrode solutions consisted of (in mM): 142 KCl, 0.5 MgCl_2_, 5 HEPES, 2 Br_2_BAPTA, and enough CaCl_2 _to produce a free Ca^2+ ^concentration of 1 μM calibrated as described previously [24]. For cell-attached recordings, membrane voltage was varied over a large range after seal formation to detect the presence of channel related fluctuations. Under these conditions, the equilibrium potential for current through a potassium selective channel would be approximately 0 mV (across the membrane). This potential is achieved when the pipette holding potential is equal to the resting potential of the hair cell (about -50 mV). The intracellular calcium concentration is unknown, but BK channels can be independently gated by voltage at large depolarizations [76]. Patches were determined to include BK channels if the channel was potassium selective, single-channel conductance exceeded 150 pS, and open probability increased with increasing depolarization. Single-channel conductance was estimated at several voltages where unitary gating events could be observed. Compared with excised patches under symmetrical K^+ ^conditions, single-channel BK conductance is lower in the cell-attached configuration, likely due to asymmetry in K^+ ^concentration across the patch or Na^+ ^block in the intact cell [77,78]. When BK channels were found at E17 to E19, patches were excised into the inside-out configuration and exposed to various free Ca^2+ ^concentrations maintaining equimolar potassium across the patch [24]. For whole-cell recordings, electrodes were filled with an internal solution containing (in mM): 112 KCl, 2 MgCl_2_, 0.1 CaCl_2_, 11 EGTA, 10 HEPES, 5 Na_2_ATP, 8 glucose and buffered to pH 7.2 with KOH. To block delayed-rectifier K^+ ^currents, 15 mM 4-aminopyridine (4-AP, Calbiochem) was added to internal solutions [16]. In all configurations, data were sampled at 10 - 20 kHz and low-pass filtered with a 4-pole Bessel filter at a 5 kHz cut-off frequency. All recordings were made at room temperature (21-24°C). Leak currents and capacitative transients were subtracted off-line.

## Authors' contributions

YL designed fluorogenic TaqMan probes, carried out quantitative PCR studies, and helped to draft early versions of data figures and manuscript text. GMA and MMM participated in study design and immunohistochemical analyses. LQL participated in the validation of gene expression probes and antibodies. MT conducted whole-cell electrophysiology experiments. RKD conceived of the study, participated in its design, conducted immunocytochemical analyses on isolated hair cells, performed patch-clamp electrophysiology experiments, and helped to draft the manuscript. All authors read and approved the final manuscript.

## Supplementary Material

Additional file 1 **Movie of confocal stack through anti-BK stained tall hair cell**. Confocal Z-stack movie is shown progressing through 17 optical sections. Two hair cell examples are show in one field. The upper right image corresponds to the right projection image in Figure 4A. Click here for file

Additional file 2 **Movie of confocal stack through anti-BK stained tall hair cell**. Confocal Z-stack movie is shown progressing through 13 optical sections. The cell shown corresponds to the left projection image in Figure 4A. Click here for file
